# Total Bee Dependence on One Flower Species Despite Available Congeners of Similar Floral Shape

**DOI:** 10.1371/journal.pone.0163122

**Published:** 2016-09-22

**Authors:** Juan P. González-Varo, F. Javier Ortiz-Sánchez, Montserrat Vilà

**Affiliations:** 1 Conservation Science Group, Dept. Zoology, University of Cambridge, Cambridge, United Kingdom; 2 Department of Integrative Ecology, Estación Biológica de Doñana (EBD-CSIC), Sevilla, Spain; 3 F.J. Ortiz-Sánchez, Universidad de Almería, ES-04120 La Cañada, Almería, Spain; Indian Institute of Science, INDIA

## Abstract

Extreme specialization is a common phenomenon in antagonistic biotic interactions but it is quite rare in mutualistic ones. Indeed, bee specialization on a single flower species (monolecty) is a questioned fact. Here, we provide multiple lines of evidence on true monolecty in a solitary bee (*Flavipanurgus venustus*, Andrenidae), which is consistent across space (18 sites in SW Iberian Peninsula) and time (three years) despite the presence of closely related congeneric plant species whose flowers are morphologically similar. The host flower (*Cistus crispus*, Cistaceae) is in turn a supergeneralist, visited by at least 85 insect species. We uncover ultraviolet light reflectance as a distinctive visual cue of the host flower, which can be a key mechanism because bee specialization has an innate basis to recognize specific signals. Moreover, we hypothesized that a total dependence on an ephemeral resource (i.e. one flower species) must lead to spatiotemporal matching with it. Accordingly, we prove that the bee’s flight phenology is synchronized with the blooming period of the host flower, and that the densities of bee populations mirror the local densities of the host flower. This case supports the ‘predictable plethora’ hypothesis, that is, that host-specialization in bees is fostered by plant species providing predictably abundant floral resources. Our findings, along with available phylogenetic information on the genus *Cistus*, suggest the importance of historical processes and cognitive constraints as drivers of specialization in bee-plant interactions.

## Introduction

Specialization on a single food resource involves a total dependence on its distribution across space and time. Although such extreme specialization is a frequent phenomenon between phytophagous insects and their host plants [1–4], it is quite rare between insect pollinators and their host flowers [5, 6]. Notably, mutualistic interactions lack a coevolutionary arms race analogous to that occurring between the plants’ secondary compounds and the herbivores’ detoxification ability [7–9]. As a consequence, most plant-pollinator interactions are characterized by non-reciprocal specialization (or asymmetry), i.e. specialist species tend to depend on generalist partners [10–12].

Bees are the world’s primary pollinators in most ecosystems [13, 14]. Unlike other taxa such as flies and butterflies, which only feed on floral resources during the adult stage, bees mainly depend on pollen and nectar to feed their larvae [15]. Nectar provides carbohydrates necessary for energy requirements, whereas pollen provides proteins necessary for larval growth. Importantly, host-plant specialization in bees is only limited to pollen, not to nectar, and physiological constraints for pollen digestion are recognized to be the main driving mechanism of specialization [16–18]. However, experiments showing successful larval development on non-host pollen have also revealed cognitive constraints [19], reflecting innate preferences (i.e. genetically based physiology) towards specific cues of the host plant (e.g. chemical cues [20]).

Host-pollen specialization in bees is classified into three major types: (*i*) polylecty, when bees collect pollen from many unrelated flower species; (*ii*) oligolecty, when bees collect pollen from species belonging to a particular plant clade, typically a genus or a few related genera; and (*iii*) monolecty, when bees collect pollen from a single host plant species [5, 21, 22]. However, most known cases of monolecty have been explained by the absence of sympatric co-flowering host-pollen congeners. In 1925, Robertson stated “… *a monolectic bee is (…) limited to one species because the genus has only one*” [21]. For decades, monolecty has been considered ‘oligolecty without choice’ [5], reflecting an absence of sympatric, synchronic host congeners. Indeed, true monolecty in the presence of co-flowering congeners has been anecdotally documented in only a handful of bee species [23–25]. Despite the potential of monolecty to add new insights into the evolutionary ecology of specialization in mutualistic interactions, there is a lack of quantitative studies–well replicated in space and time–testing hypotheses inherent to this extreme bee-plant dependence [6]. Because true monolecty entails a total dependence on an ephemeral resource (i.e. a single flower species), it must lead to (1) phenological synchronization of the bee’s flight activity period with the blooming period of the host flower, and (2) spatial concordance in bee density with host-flower density. The first hypothesis involves an evolutionary process where selection favours a phenology that coincides with that of the host flower [3, 26–28]. The second one involves an ecological process in which the density of the host flower is a major factor influencing the population density of a monolectic bee [1, 2, 28].

Here, we document the first and most comprehensive case of true monolecty, which is consistent across space (18 sites) and time (three years) despite the presence of different co-flowering (and closely-related) congeneric species. We discover ultraviolet light reflectance to be a distinctive visual cue of the host-flower. Furthermore, we tested our hypotheses concerning this extreme bee-plant dependence: (1) synchronization and (2) spatial concordance in density with the host-flower. The bee (*Flavipanurgus venustus* [Erichson, 1835], Andrenidae) is solitary and belongs to a genus that is endemic to the Iberian Peninsula (Spain and Portugal), which hosts five recognized species [15, 29]; only three records of this species were known before the present study [30]. The host flower (*Cistus crispus* L., Cistaceae, purple-flowered linage [31]) is in turn a supergeneralist (sensu [32]), visited by numerous insect species. The core distribution of this scrub species is also the Iberian Peninsula, although it marginally occurs in other areas of the western Mediterranean Basin [31].

## Results

### True monolecty across space and time

**Fig 1:**
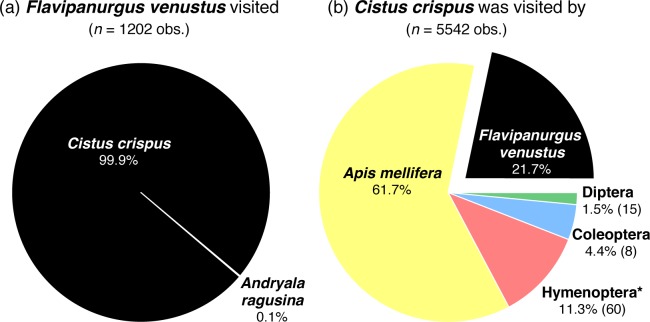
**Interaction strength (frequency, %) (a) between *Flavipanurgus venustus* bees and the flowers they visited and (b) between *Cistus crispus* flowers and the insects that visited them.** Data from three flowering seasons (2011, 2012 and 2013) in 17 studied woodland patches, based on 75 hours of observations (all data pooled). Numbers in parentheses denote the number of species of within each insect order. * Hymenoptera excluding *F*. *venustus* and *A*. *mellifera*.

**Fig 2:**
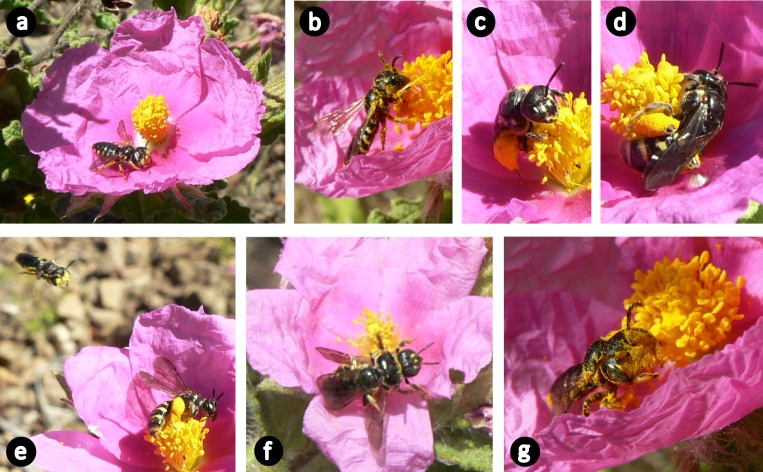
Behaviours of *Flavipanurgus venustus* on *Cistus crispus* flowers. (a) Males (unusually large head with yellow face and big mandibles) spend most of their time patrolling and defending areas of few square metres with abundant C. crispus flowers, often waiting for females landed on petals; (b) occasionally, they feed on C. crispus pollen and nectar; (c-d) females (small head with black face) spend most of their time visiting C. crispus flowers and collecting pollen loads in their scopa for provisioning the brood cells; (e) males (see top-left corner) approach females that are feeding in their territory and (f) copulate with them (notice that in the photo the wings of the male are closed while the wings of the female are open); (g) after the copula, males remain territorial waiting for other females. The body length is 8–10 mm. Notice that males and females touch both anthers and stigmas. Photos: J.P. González-Varo.

During the field surveys carried out in the 17 woodland patches and three study years, we recorded and identified more than 16,500 insect-flower interactions. Nearly 80% of these interactions were bee-flower interactions (ca. 13,000). *Flavipanurgus venustus* was recorded in 16 out of the 17 study patches (S2 Table), where it was involved in 1202 interactions, virtually 100% of which were with *C*. *crispus* flowers (see Fig 1A). Only one observation was made on a different flower species (*Andryala ragusina*, Asteraceae), likely a bee resting on its capitulum. Notably, we recorded a total of 115 flower species belonging to 38 families along the same fixed transects where we sampled pollinators (see S3 Table); the study patches had a mean of 16.0 and 15.8 flower species in the sampling periods ‘March−April’ and ‘April–May’, respectively (see details on local flower assemblages in S3 Table). During our observations, we corroborated the intimate association between the bee and *C*. *crispus*, which was not only used as food source but also as mating site (see Fig 2). Voucher specimens of *F*. *venustus* are deposited at EBD–CSIC (*n* = 51, 15 males and 36 females).

Supporting our observations, pollen analysis revealed that a mean of 97.6% (*n* = 19) of pollen grains from female scopal loads belonged to *C*. *crispus* (see S1 Appendix). This value is above the threshold (> 95%) proposed for monolecty [22]. Even on the body of males, a mean of 95.0% (*n* = 7) of pollen grains belonged to *C*. *crispus*. Some evidence suggests that the few non-*C*. *crispus* pollen grains were dropped on *C*. *crispus* flowers by domestic honeybees (*Apis mellifera* [Linnaeus, 1758]; Fig 1B) that had previously visited other flower (mostly crop and non-native) species, which were located far from our study patches (see details in S1 Appendix).

Remarkably, other *Cistus* species (*C*. *salviifolius*, *C*. *monspeliensis* and *C*. *ladanifer*) with very similar flowers were present in all study patches as main components of flower cover, overlapping phenologically with *F*. *venustus* and *C*. *crispus* (S2 Fig). All these congeneric species belong to the white-flowered lineage [31] and show a blooming peak in the ‘March−April’ sampling period (S2 Fig). However, almost all *F*. *venustus* observations (99.7%) were made during the ‘April−May’ period, when *C*. *crispus* is the most abundant flower among the *Cistus* species (S2 Fig).

**Fig 3:**
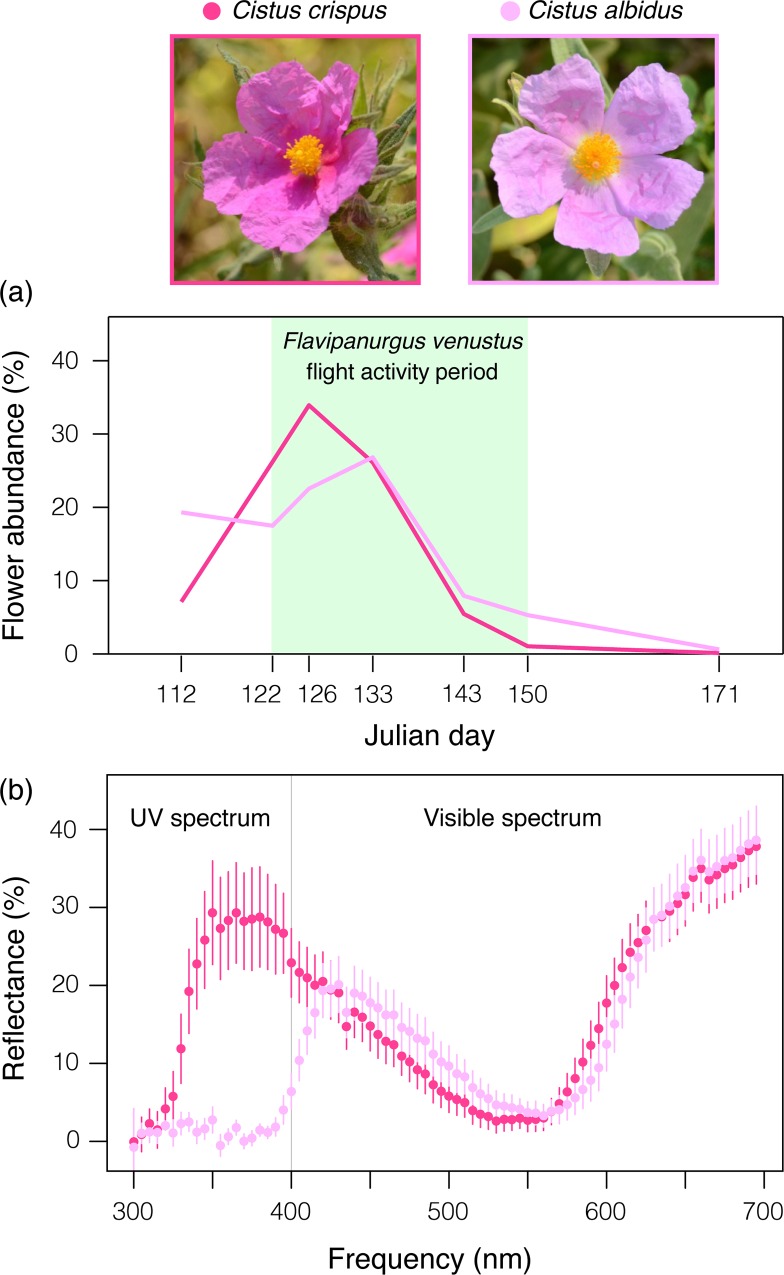
Monolecty in the presence of the closest relative species in the region. (a) Flowering phenology of the closely related *Cistus crispus* and *C*. *albidus* in a woodland patch where both co-occur (corolla diameter of 5 and 6 cm, respectively). In this site, several *Flavipanurgus venustus* bees were always observed on *C*. *crispus* flowers (*n* = 53 observations; the green area denotes the local flight activity period of the bee). (b) Reflectance (%, mean ± sd) of *C*. *crispus* and *C*. *albidus* petals (*n* = 17 flowers per species). Whereas the flowers of *C*. *crispus* reflect a large fraction of ultraviolet (UV) light, the flowers of *C*. *albidus* only reflect a negligible fraction. Note how, in the left photo, a female *F*. *venustus* is collecting *C*. *crispus* pollen in its scopa while a male (upper-left corner) is approaching for mating.

In 2013, we corroborated that *F*. *venustus* only visited *C*. *crispus* even in the presence of its closest relative species in the region, *C*. *albidus* (Fig 3A). In the studied woodland where both species co-occur, *C*. *crispus* flowers were 2.7-fold more abundant than *C*. *albidus* ones (cumulative numbers of flowers during the bee’s activity period were 1334 and 492, respectively). However, the observed frequency of visits (53 to *C*. *crispus* versus zero to *C*. *albidus;* Fig 3A) highly significantly differed from the expected frequency based on the local flower abundance (exact binomial test: *P* = 5.9 × 10^−8^). Even in the penultimate sampling survey (Julian day 150; Fig 3A), when the transect had more flowers of *C*. *albidus* (*n* = 26) than of *C*. *crispus* (*n* = 15), the seven visits recorded that day by *F*. *venustus* individuals were to *C*. *crispus*. The lack of visits to *C*. *albidus* was also corroborated in spring 2015 at this site and two newly located sites where both purple-flowered *Cistus* co-occur (S1 Fig). Interestingly, spectrometer measurements revealed that reflectance of *C*. *crispus* and *C*. *albidus* flowers greatly differs in the ultraviolet spectrum, despite reflectance is nearly identical in the visible spectrum (Fig 3B). Flowers of *C*. *crispus* reflected a large fraction of UV light, unlike those of *C*. *albidus*, which only reflected a negligible fraction.

Taken together, these findings demonstrate that *F*. *venustus* does not visit flowers (or collect pollen) of co-flowering congeneric species, even the closest relative species in the region, and that its monolecty is consistent across space and time. It is noteworthy that during the three years of study we simultaneously sampled pollinators and plant-pollinator interactions in several habitat types adjacent to the study woodland patches (grasslands, pastures, olive and orange orchards, road verges, strawberry fields, etc.), where *C*. *crispus* was absent. In such surveys, in which we recorded ~8900 plant-pollinator interactions, we never observed *F*. *venustus* (González-Varo, *unpubl*. *data*). We are thus confident that the true monolecty reported here is not the result of sampling effort concentrated in woodland habitats.

From the plant’s perspective, *C*. *crispus* was involved in 5542 of sampled interactions in which 85 insect species participated as flower visitors: 62 bees, 15 flies and 8 beetles (Fig 1B). It is therefore a supergeneralist flower (sensu [32]). The domestic honeybee accounted for 62% of all interactions (Fig 1B) followed by *F*. *venustus*, which accounted for 22% of the interactions, being the most frequent wild species visiting *C*. *crispus* flowers (Fig 1B). *C*. *crispus*–*F*. *venustus* interactions were even more frequent than the pool of the interactions with the remaining 83 flower visitor species.

### Phenological synchronization with the host flower

**Fig 4:**
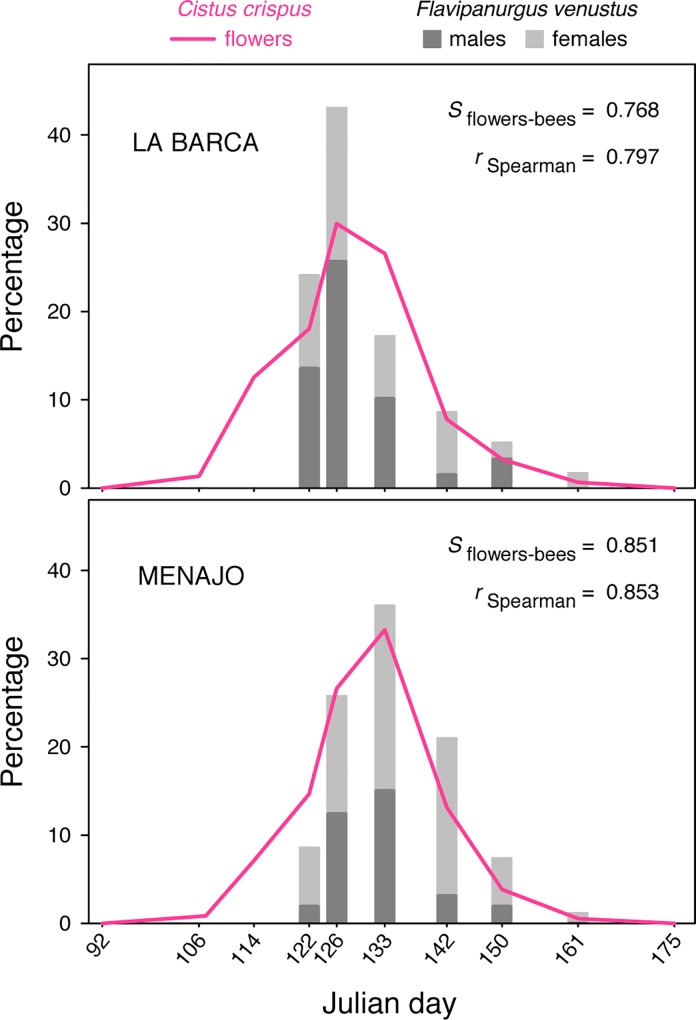
Phenological synchronization of *Flavipanurgus venutus* bees with *Cistus crispus* flowers. Data from two woodland patches periodically sampled during the spring 2013; *n* = 10 surveys in each woodland patch. *S*: Mahoro’s synchrony index; Spearman’s *r*: *P* < 0.01 in both patches.

According to our first hypothesis, the flight activity period of *F*. *venustus* was highly significantly synchronized with the flowering of *C*. *crispus* in the two woodland patches selected and monitored during 2013 (Fig 4). The Mahoro’s synchrony index (*S*_flowers–bees_) was 0.768 in La Barca and 0.851 in Menajo, which can be interpreted as phenological overlaps of 77% and 85%, respectively (Fig 4). Interestingly, in both woodland patches there was a delay between the beginning of the flowering of *C*. *crispus* and the first observation of *F*. *venustus* (Fig 4). Moreover, there was a parallel time lag of one week between the two woodland patches in reaching the phenological peak of both *C*. *crispus* flowers and *F*. *venustus* bees (Fig 4). The sites are only 6.5 km apart. This one week disparity between sites in reaching their phenological peak can be attributed to differences in tree cover (La Barca: peak on 6 May, tree cover ≈ 20%; Menajo: peak on 13 May, tree cover ≈ 40%), which influence understorey irradiance and temperature, consequently affecting bee activity and flower phenology.

### Spatial concordance with host-flower abundance

**Fig 5:**
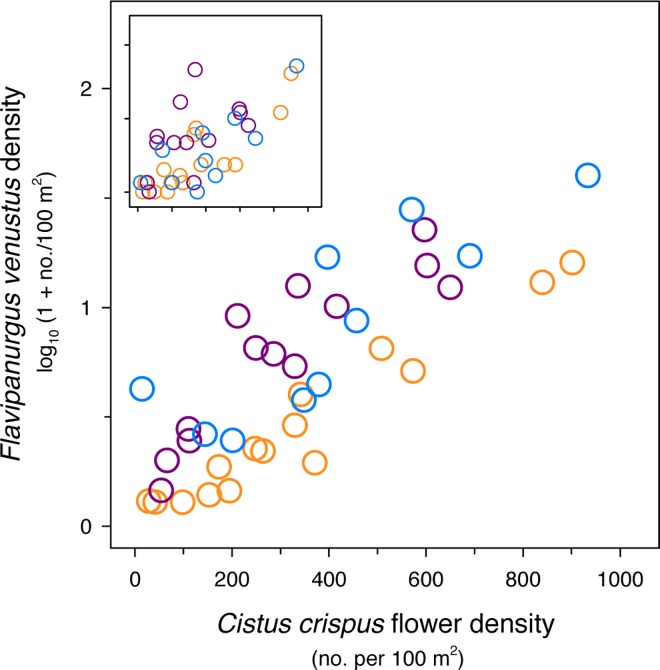
Relationship between the density of *Flavipanurgus venustus* and that of *Cistus crispus* flowers in the study woodland patches. *n*_total_ = 38 ‘woodland patch × year’ combinations; orange, purple and blue circles are 2011 (*n* = 15 patches), 2012 (*n* = 13) and 2013 (*n* = 10) data, respectively. The large panel shows bee densities predicted by the model that included the sampling date (Julian day) as covariate. The inset represents the observed bee densities (same scale as the large panel in both axes).

According to our second hypothesis, the local density of *F*. *venustus* bees mirrored that of *C*. *crispus* flowers (see Fig 5). The density of *F*. *venustus* bees was also significantly predicted by the density of *C*. *crispus* flowers (*β* = 0.286 ± 0.076, *t* = 3.74, *P* < 0.001) in the model that incorporated sampling date as a covariate (that is, ‘*bees ~ flowers + date*’: ΔAICc = 1.9, *R*^2^_LMM (*m*)_ = 0.479). In this model, the potential phenological effects associated with the sampling dates were controlled when testing for the pure effects of local flower abundance. Indeed, the sampling date had significant effects in the model (*β* = 0.230 ± 0.057, *t* = 4.05, *P* < 0.001), which is congruent with the phenology of both flowers and bees (Fig 4). Notably, this model had a better fit than the one that only incorporated flower abundance as a predictor (ΔAICc = 13.5; S4 Table). We found significant correlations between the flower densities of *C*. *crispus* at the study patches in the different study years, indicating temporal consistency in local blooms (Spearman’s rank correlations: *r*_s 2011–2012_ = 0.69, *n* = 11; *r*_s 2011–2013_ = 0.98, *n* = 8; *r*_s 2012–2013_ = 0.89, *n* = 10; all *P* ≤ 0.02).

Besides, results from models that incorporated–thus, that controlled for–the size, the woodland cover in the surrounding landscape and the geographic location of the study patches, showed that *F*. *venustus* density was significantly and consistently predicted by the density of *C*. *crispus* flowers (S4 Table). In such models, only the geographical coordinate *x* of patches had significant effects and improved model fit.

## Discussion

We have provided the most convincing evidence of true monolecty in a bee species, confirming its consistency across space and time even in the presence of different co-flowering, and morphologically similar, congeneric species. Moreover, our results clearly support our two hypotheses, namely that extreme trophic specialization on an ephemeral resource (that is, the blooming period of a single flower species) must lead to phenological synchronization and spatial concordance in density.

The lack of observed visits to flower species other than *C*. *crispus*, along with the multiple lines of evidence supporting that the non-*C*. *crispus* pollen grains found on the bodies of *F*. *venustus* individuals accounted for heterospecific pollen transfer by domestic honeybees via *C*. *crispus* shared flowers (see S1 Appendix), suggest that this monolectic bee also depends on its host flower for nectar. Although the flowers of the genus *Cistus* are characterized by their pollen reward, they also produce little quantities of nectar [33]. Yet, such relatively small nectar production in *C*. *crispus* would be compensated with abundant and reliable blooms [9].

As predicted, the flight activity period of *F*. *venustus* was highly synchronized with the blooming period of *C*. *crispus*. Yet, there is a short delay in bee activity in relation to *C*. *crispus* blooming (Fig 4), which resembles the one found by Larsson & Franzén [28] in the oligolectic bee *Andrena hattorfiana*. This lag seems to ensure enough host-flower availability when the bees emerge, being thus potentially more advantageous than a perfect synchronization (that is, *S*_flowers–bees_ ≈ 1.0). At the beginning of its blooming, *C*. *crispus* flowers are scant and sparse, thus, foraging efficiency must be low at this period because inter-flower movements must be longer and pollen rewards are expected to be poorer owing to a higher competition for them. Hence, one would expect natural selection to strongly favour the observed pattern, that is, the emergence dates of *F*. *venustus* do not coincide with the early blooming of the host flower but rather they are closer to the blooming peak. Given the extreme dependence on the host-flower species, our results raise the question as to what kind of mechanism controls the timing of emergence of this ground-nesting bee (see [15, 27, 34, 35]).

On the other hand, the density of *F*. *venustus* increased with increasing density of *C*. *crispus* flowers, supporting the prediction that host-flower abundance is a major factor influencing the population density of this monolectic bee. Indeed, the density of *C*. *crispus* flowers accounted for 48% of observed variance in density of *F*. *venustus* after controlling for differences in sampling date. This is a remarkable predictive power considering that many other factors and processes not explored in this study (e.g. habitat structure, interspecific competition, natural enemies) could potentially affect *F*. *venustus* densities. Moreover, the density of *C*. *crispus* flowers was the main predictor in models that also included landscape features and the geographical location of the study patches. Because study patches were similar in size (1.0–7.5 ha), local average bee densities (years pooled) were highly correlated with estimated bee population sizes (Pearson’s *r* = 0.89, *P* < 0.001), calculated as ‘patch size × density’ (see [28]). Our results are congruent with reported patterns of increased density of monophagous herbivorous caterpillars with increasing density of their host plants [1, 2], providing the first empirical evidence in a monophagous but mutualistic animal.

But why specialize in a single host-flower? The ‘constraint hypothesis’ of host-range evolution in bees states that specialization can be selected for if it entails higher pollen harvesting-efficiency, a larger pollen supply and/or higher pollen quality than a broader diet [36]. *Cistus crispus* may meet these three requirements since it is a dominant and predictable component of flower assemblages in the study region (accounting on average for 44% of local flower cover in the patches studied during late April−early May) and produces large amounts of pollen [37]; a ‘predictable plethora’ *sensu* Wcislo & Cane [9]. However, these features are also shared by many other *Cistus* species in the region, whose pollen is expected to be similar in nutritive terms [38]. Then, why specialize in a single floral host species when co-flowering congeneric species that seem functionally similar are available?

Although identifying the processes that drove to monolecty is beyond the scope of our study, we can discuss potential causes based on available knowledge on the evolutionary history of *Cistus* species. The genus *Cistus* radiated during the Quaternary (Pleistocene, last 2 Ma) into 21 species, 16 of which are distributed across the Mediterranean Basin [31]. Its rapid recent diversification is attributable to the establishment of the Mediterranean climate *~*3 Ma [31, 39], whose warm dry summers and cool humid winters replaced the tropical and sub-tropical climate of the Tertiary [40]. Such climatic changes caused the decline of the lauroid subtropical forests in the Mediterranean Basin and the extinction of many members of its flora [40], but led to the rise of new vegetation types dominated by scrub species belonging to a few families (mainly Cistaceae, Lamiaceae, Fabaceae) that radiated during the Pleistocene [41]. The divergence between *C*. *crispus* and *C*. *albidus*, the two purple-flowered *Cistus* species distributed in the region, is dated between 1−2 Ma [31, 39]. Thereby, the origin of monolecty in *F*. *venustus* may be framed in the glacial and interglacial periods of the Pleistocene, under a context of high geographical isolation and low co-occurrence among *Cistus* species, which might have favoured the extreme specialization of *F*. *venustus* on *C*. *crispus* flowers in the lowlands of the south–western Iberian Peninsula.

Independent of the origin of monolecty on *C*. *crispus*, we argue that cognitive constraints driven by neurological adaptations for host-flower recognition is the most plausible mechanism that nowadays limits *F*. *venustus* feeding on other *Cistus* flowers [16, 19, 36]. Praz and collaborators [19] demonstrated by means of rearing experiments how the preference of a specialist bee is innate and not the result of imprinting with pollen supplied by the mother. Innate preference involves a genetic basis to recognize a specific cue of the host flower (olfactory, visual or both) [19, 42]. The flowers of *C*. *crispus* reflect a huge fraction of ultraviolet light unlike the flowers of *C*. *albidus* (Fig 3B) and those of the white-flowered *C*. *monspeliensis* and *C*. *salviifolius* [43]. Thus, ultraviolet light may be a major cue by which *F*. *venustus* recognizes *C*. *crispus* as its host-flower [44]. This does not necessarily mean that ultraviolet light is the only cue used by the bee, as recent studies have also shown the important role of olfactory cues for host recognition in bees [45, 46]. However, we discard physiological limitations for pollen digestion as the underlying mechanism of monolecty because such constraints typically occur at higher taxonomic levels, when the non-host pollen belongs to different genera or families [17, 47]. Indeed, differences in pollen structure and nutritional composition are usually small within a genus, as found within *Cistus* [38, 48]. Remarkably, the pollen of *C*. *crispus* and *C*. *albidus* are indistinguishable, even when using scanning electron microscopy [48].

In accordance with mutualistic network studies [10–12], this bee-flower interaction is hugely asymmetric in terms of the number of interacting partners, with *F*. *venustus* representing just one out of the 85 flower visitor species interacting with *C*. *crispus*. Nevertheless, asymmetry was much lower in terms of interaction strength, as *F*. *venustus* accounted for 22% of *C*. *crispus* interactions, yet represented a higher percentage than the pool of the interactions with the other wild insect species (17%; Fig 1B). Visits to *C*. *crispus* flowers were indeed dominated by the honeybee (62%), as already found for other insect-pollinated species in the study region due to the widespread presence of managed hives [49]. As the contemporary superabundance of the honeybee is man-mediated, we envision a past scenario where *F*. *venustus* acted as the most frequent visitor to *C*. *crispus* flowers (Fig 1B). During their visits, *F*. *venustus* bees touch both anthers and stigmas of *C*. *crispus* flowers, thereby, promoting pollination. Our findings thus indicate a strong and common mutualistic interaction between an extreme specialist and a supergeneralist. This finding represents an exception to the general observation in mutualistic networks, where strong interactions tend to occur between generalists [50, 51]. It also provides a nice example of non-existent interactions (i.e. those between *F*. *venustus* and the other *Cistus* species) that would be predicted to occur on the basis of morphological traits or phylogenetic distance (false positives) [52].

In conclusion, our study uncovers in an unprecedented way a case of true monolecty in bees, adding new insights into the evolutionary ecology of extreme specialization in mutualistic interactions while raising several intriguing questions. What mechanisms control the timing of bee emergence, and thus phenological synchronization with the host-plant? What is the whole distributional range of *F*. *venustus* in relation to that of *C*. *crispus*? What is the degree of trophic specialization in the other *Flavipanurgus* species? And, how does the phylogeny of this genus compare with the divergence dates of *Cistus* species? We lack knowledge about the natural history, ecology and conservation status of most extant ca. 20,000 bee species [14, 15], especially in diversity hotspots like the Iberian Peninsula [53, 54]. Although contemporary communities may only provide a poor representation of the originals [55], they still offer us the opportunity to act as–using Janzen’s words [56]–“*archivists for what were once powerful and complex interactions*”.

## Materials and Methods

### Sampling pollinators and plant-pollinator interactions

During the springs of 2011, 2012 and 2013, we conducted intensive field surveys to sample pollinators and plant-pollinator interactions in 17 Mediterranean woodland patches located in Huelva province, SW Spain. Such patches were small woodland remnants (1.0–7.5 ha; median = 2.2 ha; S1 Table) located in agricultural landscapes, and extending over an area of ca. 70 km in longitude × 20 km in latitude (S1 Fig). All pairwise distances between patches were > 2 km (up to 68 km), except for two patches separated by 130 m. The climate in this region is typically Mediterranean, with warm dry summers and cool humid winters; mean annual precipitation is 525 mm and January and July temperatures average 11°C and 26°C, respectively [57].

Sampling consisted of capturing/recording all insect pollinators observed along two fixed belt transects (1 m × 150 m during 15 min) randomly located in each woodland patch, aiming to cover a random representation of local flower assemblages. An insect was considered a pollinator if it visited a flower and touched its sexual parts (anthers and stigmas). Captured specimens were identified by F.J. Ortiz-Sánchez. Pollinator specimens are deposited at EBD-CSIC. In the same transects, we also recorded the number and identity of all open flowers. Data from the two transects were pooled and treated as one 300 m^2^ belt transect in subsequent statistical analyses. Transects were sampled in four sampling rounds, two from late March to late April (‘March–April’, hereafter) and the other two from late April to mid/late May (‘April–May’, hereafter). Sampling was conducted between 10:00 and 17:00, on sunny or slightly cloudy days with little or no wind (wind speed < 20 km h^−1^). Whenever possible, the two rounds belonging to the same sampling period were conducted at a different time of day; ‘morning’ (~10:00–13:00) or afternoon (~14:00–17:00). During the three years of study we sampled a total of 298 transects in 38 ‘patch × year’ combinations (15 in 2011, 13 in 2012 and 10 in 2013) accounting for a total of 75 hours of observation (see details in S1 Table).

### Corroborating monolecty with pollen analysis

We analysed the pollen loads of 26 *F*. *venustus* bees (7 males and 19 females captured during the ‘April−May’ period) from four sites (5–10 individuals per site) that were individually placed in clean kill-vials. To obtain pollen samples, small cubes (ca. 3 × 3 × 1 mm) of fuchsine-stained gelatine were rubbed over the female scopa or male body and mounted on glass slides (see S1 Appendix), following [58]. Pollen grains were identified in the microscope by experts (David Navarro and Rut Puigdemunt, Universitat Autònoma de Barcelona). Pollinator specimens and pollen slides are deposited at EBD-CSIC. Notably, the pollen of *C*. *crispus* is distinguishable from the white-flowered *Cistus* species locally present in the studied patches [48]; see below.

### Monolecty in the presence of the closest relative flower

Other *Cistus* species besides *C*. *crispus* occurred in the 17 study patches, all of them belonging to the white-flowered lineage [31]. The only *Cistus* species belonging to the purple-flowered lineage in the study region is *C*. *albidus*, which has nearly identical flowers to *C*. *crispus* (Fig 3A) and is phylogenetically its closest relative species in the southern Iberian Peninsula [31]. Both purple-flowered species rarely co-occur in the region. Indeed, *C*. *albidus* was not present in any of the 17 study patches. In 2013, we located an additional woodland patch within our study area where *C*. *crispus* and *C*. *albidus* co-occur (S1 Fig), in order to corroborate monolecty even in the presence of *C*. *albidus*. We monitored flower abundance and *F*. *venustus* visits along a fixed transect belt (370 m × 2 m) during the whole flowering phenology of *C*. *crispus*, in seven surveys from 22 April to 10 June. We used exact binomial test to assess whether the frequency of visits to each purple-flowered *Cistus* species significantly differed from the expected frequency based on their local abundance.

In spring 2015, we surveyed this site and two newly located sites where *C*. *albidus* was also present in order to corroborate the monolecty of *F*. *venustus* (S1 Fig). It is noteworthy that *C*. *albidus* is very abundant in eastern Iberian Peninsula (unlike *C*. *crispus*), where recognized Spanish bee experts have never observed *F*. *venustus* bees despite decades of fieldwork (F.J. Ortiz-Sánchez & J. Bosch; *personal communication*).

### The cue of the host-flower: reflectance spectra

*Flavipanurgus venustus* bees were always observed on *C*. *crispus* even in the presence of *C*. *albidus*, the closest relative flower in the region (see Results). Although both flower species are nearly identical to the human eye (Fig 3), they may differ in the UV spectrum, which is visible to bees and thus an important cue [44]. In spring 2015, we used an Ocean Optics USB-2000 spectrometer and a Top Sensor System deuterium–halogen DH-2000 lamp as a standardized light source (DT-MINI-GS-2) to measure the reflectance spectra of *C*. *crispus* and *C*. *albidus* flowers, in order to assess differences in their colours (see details in [59]). We averaged reflectance data in 5-nm-wide spectral intervals (~14 data points per interval) over the range of 300–700 nm. We measured the reflectance of 17 petals per species, each from a different individual plant. We then calculated a mean ± sd reflectance spectrum for each species.

### Phenological synchronization with the host flower

In 2013, we conducted periodical surveys during the whole flowering period of *C*. *crispus* in two study patches in order to assess the degree of phenological synchronization between the bee (*F*. *venustus*) and its host flower. The selected study sites were La Barca and Menajo (6.5 km apart), where the densities of *F*. *venustus* in 2011 and 2012 were consistently intermediate (La Barca 11 and 7 bees per 100 m^2^) and high (Menajo: 40 and 45 bees per 100 m^2^) in relation to the densities recorded during these years in all patches (mean 6 bees per 100 m^2^). Sampling consisted of counting all *F*. *venustus* bees and *C*. *crispus* flowers along two fixed belt transects (1 m × 150 m during 15 min) as those described above; data from both transects were pooled for data analysis (300 m^2^). Transects were sampled weekly or biweekly, in ten surveys from 2 April to 24 June carried out between 12:00 h and 14:00 h, once *F*. *venustus* reached its maximum activity period.

We applied the Mahoro’s synchrony index [60] (*S*_*i*_ = ½ [2 –Σ |*y*_*bees*, *j*_−*y*_*flowers*, *j*_|]) to quantify the phenological synchronization between the relative abundances of *F*. *venustus* bees (*y*_*bees*_) and *C*. *crispus* flowers (*y*_*flowers*_) in each sampling survey (*j*). The index ranges from 0 (no phenological overlap) to 1 (perfect phenological matching). Moreover, we used Spearman’s rank correlation to test the significance of the relationship between *y*_*bees*, *j*_ and *y*_*flowers*, *j*_.

### Spatial concordance with host-flower density

We used the data from the intensive field surveys conducted during the springs of 2011, 2012 and 2013 (described above) to test for spatial concordance between the density of *F*. *venustus* and that of *C*. *crispus* flowers. Overall, our dataset included 17 woodland patches that were sampled in three different years leading to 38 ‘site × year’ combinations (S1 Table). We first extracted the counts of *C*. *crispus* flowers and *F*. *venustus* along all 300m^2^ belt transects. We then selected only data belonging to the sampling period ‘April–May’ because it is the period that included the flight phenology of *F*. *venustus* (99.7% of observations, see Results) and the flowering peak of *C*. *crispus* (S2 Fig). Moreover, of the two sampling rounds carried out during the period ‘April–May’ we only selected data belonging to the ‘afternoon’ round, when the activity of the bee was greatest. As mentioned, *F*. *venustus* typically reached its maximum activity period after 12:00 h, when temperatures were usually above 25°C. We exclude the ‘morning’ rounds because many of them were carried between 10:00 and 12:00, before bees were locally observed or before they reached the maximum activity. The resulting data subset (i.e. ‘afternoon round’ of the ‘April–May’ period) accounted for ~65% of the total *F*. *venustus* counts. As this subset only includes one sampling round, data for subsequent analyses consisted of a single 300 m^2^ belt transect per patch and year.

We modelled the abundance of *F*. *venustus* in response to the abundance of *C*. *crispus* flowers (predictor variable) using linear mixed models (LMMs) that included ‘site’ as a random factor to account for the woodland patches that were re-sampled in different years. Bee counts were log_10_-transformed to reduce positive skew. Considering the phenology of the study system, any spatial pattern between the abundances of the host-flower and the bee could actually be mirroring a temporal pattern if some patches were sampled closer to their phenological peak than others (Fig 3). Sampling dates ranged between Julian days 115 and 143 (mean = 129.2, SD = 8.2 days); thus, low values represent the beginning of the phenology of both flowers and bees while high values represent the peak (Fig 3). In order to control for differences in sampling date between patches, we built an additional LMM that incorporated the Julian day of the sampling survey as a fixed factor (correlation flower abundance–Julian day: Pearson’s *r* = 0.257, *P* = 0.12). Model fit was evaluated according to the Akaike Information Criterion for small simple sizes (AICc) [61] and marginal *R*^2^ values (*R*^2^_LMM (*m*)_, that is, the variance explained by the fixed effects variables) [62]. LMMs were fitted with R (v. 3.0.2) [63] using the packages *lme4* (v. 1.0–5) [64] and *lmertest* (v. 2.0–3) [65].

Furthermore, because our study sites were in a fragmented area, we tested whether patch size and woodland cover within a 1-km radius around our study patches influenced the density of *F*. *venustus*. For this purpose, we built additional LMMs including both variables as predictors (S4 Table). We also built additional LMMs including the geographical coordinates (*x*, *y*) of the study patches as predictor variables in order to check for any possible spatial trend (S4 Table).

### Ethics statement

Pollinator sampling was framed within the STEP project (*Status and Trends of European Pollinators*; http://www.step-project.net/), with the consent by the Andalusian Regional Government. The fieldwork was carried out on private land with the knowledge and consent of the owners.

## Supporting Information

S1 Appendix Analysis of pollen loads of *Flavipanurgus venustus* bees. (PDF)

S1 Fig Geographic location of both the study region in the Iberian Peninsula and the studied sites within the study area. (PDF)

S2 Fig Flowers of the more abundant *Cistus* species in the studied woodland patches and density of *F*. *venustus* bees and *Cistus* flowers in the two sampling periods. (PDF)

S1 File Data on abundances of *Flavipanurgus venustus* bees and flowers of different *Cistus* species in the study patches (per 300-m transect) and at different sampling periods (May–April’ and ‘April–May’). (CSV)

S2 File Data on reflectance (%) of *Cistus crispus* and *Cistus albidus petals*. (CSV)

S3 File Data on two woodland patches periodically sampled during the spring 2013 to assess phenological synchronization of *Flavipanurgus venutus* bees with *Cistus crispus* flowers. (CSV)

S4 File Dataset used to run the LMM models predicting the density of *Flavipanurgus venustus* bees. (CSV)

S1 Table Characteristics of the 17 study woodland patches studied. (PDF)

S2 Table Observed occurrence of *Flavipanurgus venustus* bees. (PDF)

S3 Table List of the most common flower species (and family names) recorded across the study patches in the sampling periods ‘May–April’ and ‘April–May’. (PDF)

S4 Table LMM models predicting the density of *Flavipanurgus venustus* bees. (PDF)

## References

[1] Kéry M, Matthies D, Fischer M. The effect of plant population size on the interactions between the rare plant *Gentiana cruciata* and its specialized herbivore *Maculinea rebeli*. J Ecol. 2001;89(3):418–27. 10.1046/j.1365-2745.2001.00550.x

[2] Krauss J, Steffan-Dewenter I, Müller CB, Tscharntke T. Relative importance of resource quantity, isolation and habitat quality for landscape distribution of a monophagous butterfly. Ecography. 2005;28(4):465–74. 10.1111/j.0906-7590.2005.04201.x

[3] van Asch M, Visser ME. Phenology of forest caterpillars and their host trees: the importance of synchrony. Annu Rev Entomol. 2007;52:37–55. 10.1146/annurev.ento.52.110405.091418 . 16842033

[4] Schweiger O, Settele J, Kudrna O, Klotz S, Kühn I. Climate change can cause spatial mismatch of trophically interacting species. Ecology. 2008;89(12):3472–9. 10.1890/07-1748.1 19137952

[5] Cane JH, Sipes S. Characterizing floral specialization by bees: analytical methods and a revised lexicon for oligolecty In: Waser NM, Ollerton J, editors. Plant-pollinator interactions: from specialization to generalization. Chicago: University of Chicago Press; 2006 p. 99–122.

[6] Minckley RL, Roulston TH. Characterizing floral specialization by bees: analytical methods and a revised lexicon for oligolecty In: Waser NM, Ollerton J, editors. Plant-pollinator interactions: from specialization to generalization. Chicago: Univ. Chicago Press; 2006 p. 69–98.

[7] Ehrlich PR, Raven PH. Butterflies and plants: a study in coevolution. Evolution. 1964;18(4):586–608. 10.2307/2406212

[8] Mitter C, Farrell B, Futuyma DJ. Phylogenetic studies of insect-plant interactions: Insights into the genesis of diversity. Trends Ecol Evol. 1991;6(9):290–3. 10.1016/0169-5347(91)90007-K 21232484

[9] Wcislo WT, Cane JH. Floral resource utilization by solitary bees (Hymenoptera: Apoidea) and exploitation of their stored foods by natural enemies. Annu Rev Entomol. 1996;41(1):257–86. 10.1146/annurev.en.41.010196.001353 . 15012330

[10] Bascompte J, Jordano P, Melián CJ, Olesen JM. The nested assembly of plant-animal mutualistic networks. Proc Natl Acad Sci USA. 2003;100(16):9383–7. 10.1073/pnas.1633576100 12881488 PMC170927

[11] Vázquez DP, Aizen MA. Asymmetric specialization: a pervasive feature of plant-pollinator interactions. Ecology. 2004;85(5):1251–7. 10.1890/03-3112

[12] Thébault E, Fontaine C. Stability of ecological communities and the architecture of mutualistic and trophic networks. Science. 2010;329(5993):853–6. 10.1126/science.1188321 . 20705861

[13] Neff JL, Simpson BB. Bees, pollination systems and plant diversity In: LaSalle J, Gauld ID, editors. Hymenoptera and biodiversity. Wallingford: CAB International; 1993 p. 143–67.

[14] Winfree R. The conservation and restoration of wild bees. Ann N Y Acad Sci. 2010;1195:169–97. 10.1111/j.1749-6632.2010.05449.x . 20536823

[15] Michener C. The Bees of the World, 2nd edition Baltimore and London: Johns Hopkins University Press; 2007.

[16] Williams NM. Use of novel pollen species by specialist and generalist solitary bees (Hymenoptera: Megachilidae). Oecologia. 2003;134(2):228–37. 10.1007/s00442-002-1104-4 . 12647164

[17] Praz CJ, Müller A, Dorn S. Specialized bees fail to develop on non-host pollen: do plants chemically protect their pollen? Ecology. 2008;89(3):795–804. 10.1890/07-0751.1 18459342

[18] Sedivy C, Müller A, Dorn S. Closely related pollen generalist bees differ in their ability to develop on the same pollen diet: evidence for physiological adaptations to digest pollen. Funct Ecol. 2011;25(3):718–25. 10.1111/j.1365-2435.2010.01828.x

[19] Praz CJ, Müller A, Dorn S. Host recognition in a pollen-specialist bee: evidence for a genetic basis. Apidologie. 2008;39(5):547–57. 10.1051/apido:2008034

[20] Milet-Pinheiro P, Herz K, Dötterl S, Ayasse M. Host choice in a bivoltine bee: how sensory constraints shape innate foraging behaviors. BMC Ecol. 2016;16(1):1–12. 10.1186/s12898-016-0074-z 27068328 PMC4828851

[21] Robertson C. Heterotropic bees. Ecology. 1925;6:412–36.

[22] Müller A, Kuhlmann M. Pollen hosts of western palaearctic bees of the genus *Colletes* (Hymenoptera: Colletidae): the Asteraceae paradox. Biol J Linn Soc. 2008;95(4):719–33. 10.1111/j.1095-8312.2008.01113.x

[23] Houston T, Lamont B, Radford S, Errington S. Apparent mutualism between *Verticordia nitens* and *V*. *aurea* (Myrtaceae) and their oil-ingesting bee pollinators (Hymenoptera, Colletidae). Aust J Bot. 1993;41(3):369–80. 10.1071/BT9930369.

[24] Holland J, Lanza J. Geographic variation in the pollination biology of *Passiflora lutea* (Passifloraceae). J Ark Acad Sci. 2008;26:32–6.

[25] Kuhlmann M, Timmermann K. Nest architecture of the monolectic South African solitary bee, *Samba* (*Prosamba*) *spinosa* Eardley (Hymenoptera: Apoidea: Melittidae). Afr Entomol. 2011;19(1):141–5. 10.4001/003.019.0112

[26] Minckley RL, Wcislo WT, Yanega D, Buchmann SL. Behavior and phenology of a specialist bee (*Dieunomia*) and sunflower (*Helianthus*) pollen availability. Ecology. 1994;75(5):1406–19. 10.2307/1937464

[27] Danforth BN. Emergence dynamics and bet hedging in a desert bee, *Perdita portalis*. Proc R Soc Lond, Ser B: Biol Sci. 1999;266(1432):1985–94. 10.1098/rspb.1999.0876

[28] Larsson M, Franzén M. Estimating the population size of specialised solitary bees. Ecol Entomol. 2008;33(2):232–8. 10.1111/j.1365-2311.2007.00956.x

[29] Warncke K. Ergänzende Untersuchungen an Bienen der Gattungen Panurgus und Melitturga/Andreninae, Apidae, vor allem aus dem turkischen Raum. Boll Mus Civ Stor Nat Vene. 1987;36:75–107.

[30] Patiny S. Atlas of the European Bees: genus Flavipanurgus. STEP Project, Atlas Hymenoptera Mons, Gembloux2012. Available from: http://www.zoologie.umh.ac.be//hymenoptera/page.asp?ID=24.

[31] Guzmán B, Lledo MD, Vargas P. Adaptive radiation in Mediterranean *Cistus* (Cistaceae). PLoS One. 2009;4(7):e6362 10.1371/journal.pone.0006362 19668338 PMC2719431

[32] Olesen JM, Eskildsen LI, Venkatasamy S. Invasion of pollination networks on oceanic islands: importance of invader complexes and endemic super generalists. Divers Distrib. 2002;8(3):181–92. 10.1046/j.1472-4642.2002.00148.x

[33] Herrera J. Nectar secretion patterns in southern Spanish Mediterranean scrublands. Israel Journal of Botany. 1985;34(1):47–58. 10.1080/0021213X.1985.10677008

[34] Kemp WP, Bosch J. Postcocooning emperatures and diapause in the alfalfa pollinator *Megachile rotundata* (Hymenoptera: Megachilidae). Physiology, Biochemistry, and Toxicology. 2001;94(2):244–50.

[35] Schäffler I, Dötterl S. A day in the life of an oil bee: phenology, nesting, and foraging behavior. Apidologie. 2011;42(3):409–24. 10.1007/s13592-011-0010-3

[36] Sedivy C, Praz CJ, Muller A, Widmer A, Dorn S. Patterns of host-plant choice in bees of the genus Chelostoma: the constraint hypothesis of host-range evolution in bees. Evolution. 2008;62(10):2487–507. 10.1111/j.1558-5646.2008.00465.x . 18637958

[37] Bosch J. Floral biology and pollinators of three co-occurring *Cistus* species (Cistaceae). Bot J Linn Soc. 1992;109(1):39–55. 10.1111/j.1095-8339.1992.tb00257.x

[38] Petanidou T, Vokou D. Pollination and pollen energetics in Mediterranean ecosystems. Am J Bot. 1990;77(8):986–92. 10.2307/2444569

[39] Fernández-Mazuecos M, Vargas P. Ecological rather than geographical isolation dominates Quaternary formation of Mediterranean *Cistus* species. Mol Ecol. 2010;19(7):1381–95. 10.1111/j.1365-294X.2010.04549.x . 20196815

[40] Mai DH. Development and regional differentiation of the European vegetation during the Tertiary. Plant Syst Evol. 1989;162(1–4):79–91. 10.1007/BF00936911

[41] Herrera CM. Historical effects and sorting processes as explanations for contemporary ecological patterns: character syndromes in Mediterranean woody plants. Am Nat. 1992;140(3):421–46. 10.2307/2462775

[42] Lunau K. Innate Flower Recognition in Bumblebees (Bombus terrestris, B. lucorum; Apidae): Optical Signals from Stamens as Landing Reaction Releasers. Ethology. 1991;88(3):203–14. 10.1111/j.1439-0310.1991.tb00275.x

[43] Arnold SEJ, Faruq S, Savolainen V, McOwan PW, Chittka L. FReD: The Floral Reflectance Database—A Web Portal for Analyses of Flower Colour. PLoS One. 2010;5(12):e14287 10.1371/journal.pone.0014287 21170326 PMC3000818

[44] Chittka L, Shmida A, Troje N, Menzel R. Ultraviolet as a component of flower reflections, and the colour perception of Hymenoptera. Vision Res. 1994;34(11):1489–508. 8023461 10.1016/0042-6989(94)90151-1

[45] Schäffler I, Steiner KE, Haid M, van Berkel SS, Gerlach G, Johnson SD, et al Diacetin, a reliable cue and private communication channel in a specialized pollination system. Scientific Reports. 2015;5:12779 10.1038/srep12779 http://www.nature.com/articles/srep12779—supplementary-information. 26245141 PMC4526864

[46] Milet-Pinheiro P, Ayasse M, Dobson HEM, Schlindwein C, Francke W, Dötterl S. The chemical basis of host-plant recognition in a specialized bee pollinator. J Chem Ecol. 2013;39(11):1347–60. 10.1007/s10886-013-0363-3 24233444

[47] Haider M, Dorn S, Müller A, Kudo G. Intra- and interpopulational variation in the ability of a solitary bee species to develop on non-host pollen: implications for host range expansion. Funct Ecol. 2013;27(1):255–63. 10.1111/1365-2435.12021

[48] Civeyrel L, Leclercq J, Demoly J-P, Agnan Y, Quèbre N, Pélissier C, et al Molecular systematics, character evolution, and pollen morphology of *Cistus* and *Halimium* (Cistaceae). Plant Syst Evol. 2011;295(1–4):23–54. 10.1007/s00606-011-0458-7

[49] González-Varo JP, Arroyo J, Aparicio A. Effects of fragmentation on pollinator assemblage, pollen limitation and seed production of Mediterranean myrtle (*Myrtus communis*). Biol Conserv. 2009;142(5):1058–65. 10.1016/j.biocon.2009.01.017

[50] Vázquez DP, Melián CJ, Williams NM, Blüthgen N, Krasnov B, Poulin R. Species abundance and asymmetric interaction strength in ecological networks. Oikos. 2007;116(7):1120–7. 10.1111/j.2007.0030-1299.15828.x

[51] Gilarranz LJ, Pastor JM, Galeano J. The architecture of weighted mutualistic networks. Oikos. 2012;121(7):1154–62. 10.1111/j.1600-0706.2011.19592.x

[52] González-Varo JP, Traveset A. The labile limits of forbidden interactions. Trends Ecol Evol. 2016;31(9):700–10. 10.1016/j.tree.2016.06.009 27471077

[53] Ortiz-Sánchez FJ. Lista actualizada de las especies de abejas de España (Hymenoptera: Apoidea: Apiformes). Bol Soc Entomol Arag. 2011;49:265–81.

[54] Nieto A, Roberts SPM, Kemp J, Rasmont P, Kuhlmann M, García-Criado M, et al. European Red List of bees. Luxembourg: 2014.

[55] González-Varo JP, Biesmeijer JC, Bommarco R, Potts SG, Schweiger O, Smith HG, et al Combined effects of global change pressures on animal-mediated pollination. Trends Ecol Evol. 2013;28(9):524–30. 10.1016/j.tree.2013.05.008 . 23746938

[56] Janzen DH. Two patterns of pre-dispersal seed predation by insects on Central American deciduous forest trees In: Burley J, Styles BT, editors. Tropical trees Variation, breeding and conservation Linn Soc Symp Ser 2. London: Academic Press; 1976 p. 179–88.

[57] A.E.M.E.T. Valores climatológicos normales. Huelva, Ronda Este 2015. Available from: http://www.aemet.es/es/serviciosclimaticos/datosclimatologicos/valoresclimatologicos.

[58] Bosch J, Gonzalez AM, Rodrigo A, Navarro D. Plant-pollinator networks: adding the pollinator's perspective. Ecol Lett. 2009;12(5):409–19. 10.1111/j.1461-0248.2009.01296.x . 19379135

[59] Valido A, Schaefer HM, Jordano P. Colour, design and reward: phenotypic integration of fleshy fruit displays. J Evol Biol. 2011;24(4):751–60. 10.1111/j.1420-9101.2010.02206.x 21255176

[60] Mahoro S. Individual flowering schedule, fruit set, and flower and seed predation inVaccinium hirtumThunb. (Ericaceae). Can J Bot/Rev Can Bot. 2002;80(1):82–92. 10.1139/b01-136

[61] Burnham KP, Anderson DR. Model Selection and Multimodel Inference: A Practical-Theoretic Approach, 2nd edn. New York, Berlin, Heidelberg: Springer; 2002.

[62] Nakagawa S, Schielzeth H, O'Hara RB. A general and simple method for obtaining *R*2 from generalized linear mixed-effects models. Methods Ecol Evol. 2013;4(2):133–42. 10.1111/j.2041-210x.2012.00261.x

[63] Team RDC. R: a Language and Environment for Statistical Computing. Vienna, Austria: R Fundation for Statistical Computing; 2013.

[64] Bates D, Maechler M, Bolker B. Bates, D., Maechler, M. & Bolker, B. lme4: linear mixed-effects models using S4 classes. 2013.

[65] Kuznetsova A, Brockhoff PB, Christensen RHB. lmerTest: tests for random and fixed effects for linear mixed effect models (lmer objects of lme4 package) Version 2.0–6. 2012.

