# Supplementary material for: Total Bee Dependence on One Flower Species Despite Available Congeners of Similar Floral Shape
Source: PLoS One. 2016 Sep 22;11(9):e0163122. doi: 10.1371/journal.pone.0163122 (PMC5033463; doi:10.1371/journal.pone.0163122)
Supplement: S1 Appendix — (PDF) [file pone.0163122.s001.pdf]

S1 Appendix. Analysis of pollen loads of *Flavipanurgus venustus* bees (Fig A) revealed that *Cistus crispus* accounted an average of 97.6% and 95% of pollen grains sampled from female scopa and male body, respectively. Most of non-*C. crispus* pollen grains in female samples belonged to *Fragaria* × *ananassa* (43.6%), *Acacia* spp. (13.2%), *Artemisia* spp. (11.3%), *Melilotus*–type (11.1%), *Galactites* spp. (7.7%) and *Carpobrotus* spp. (5.1%). Three major evidence lines strongly suggest that the presence of non-*C. crispus* pollen grains mostly resulted from inter-insect pollen transfer via shared flowers [*see* 1]. Fist, the honeybee (*Apis mellifera*) accounted for most of the visits to *C. crispus* flowers (Fig. 1). Second, honeybees are known to be the main visitor of crop [2] and non-native flowers [3] (JPGV *personal observations*). And third, the greenhouses of strawberries (*Fragaria* × *ananassa*) and the non-native individual plants of *Acacia* spp. and *Carpobrotus* spp. are far from our study patches (from hundreds of meters to kilometers), distances that are typically covered by the honeybee [4] but overcome the foraging ranges of small solitary bees like *Flavipanurgus venustus* [5].

Fig A (a) Small cubes of fuchsine-stained gelatine being rubbed over a female scopa; (b) two photos (different zoom) showing microscope view of pollen load samples dominated by *Cistus crispus* pollen; (c) percentage of pollen grains of *C. crispus* (red dots in boxplot represent mean values) in samples obtained from female scopa and male body (overall average 96.8%).

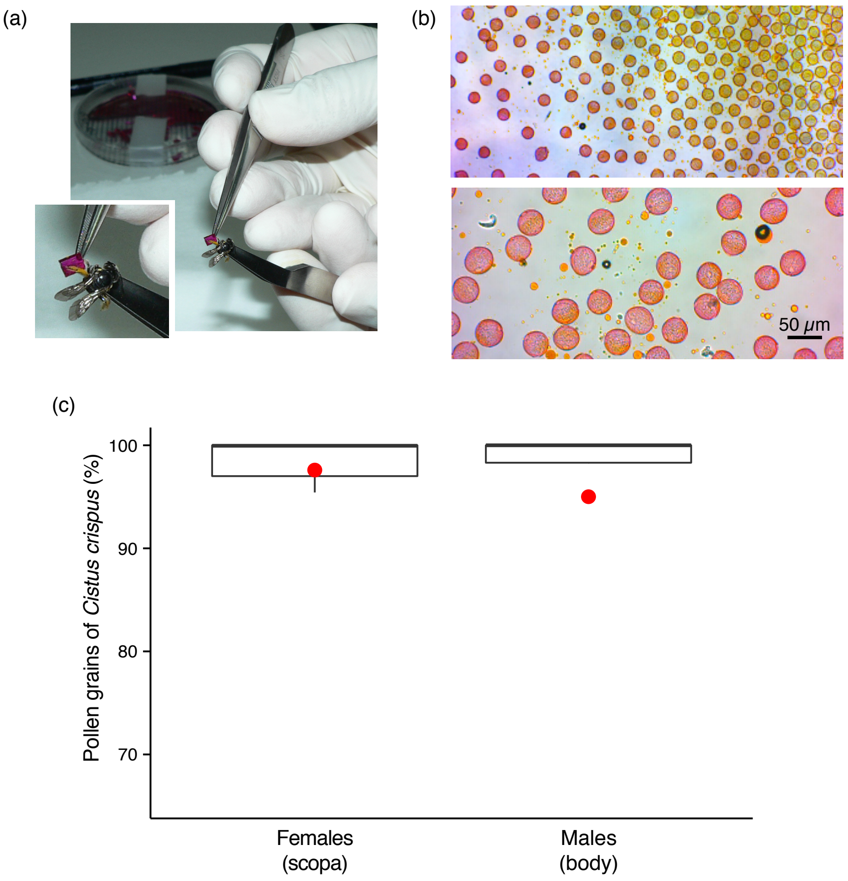

[1] Fang Q & Huang SQ (2013) A directed network analysis of heterospecific pollen transfer in a biodiverse community. *Ecology*, 94, 1176–1185.

[2] Garibaldi LA, Steffan-Dewenter I, Winfree R, Aizen MA *et al.* (2013) Wild pollinators enhance fruit set of crops regardless of honey bee abundance. *Science* 339, 1608–1611.

[3] Morales C & Aizen MA (2002) Does invasion of exotic plants promote invasion of exotic flower visitors? A case study from the temperate forests of the southern Andes. *Biol. Invas.* 4, 87–100.

[4] Steffan-Dewenter I & Kuhn A (2003) Honeybee foraging in differentially structured landscapes. *Proc. R. Soc. Lond. B* 270, 569–575.

[5] Gathmann A & Tscharntke T (2002) Foraging ranges of solitary bees. *J. Anim. Ecol.* 71, 757–764.
