# Supplementary material for: Total Bee Dependence on One Flower Species Despite Available Congeners of Similar Floral Shape
Source: PLoS One. 2016 Sep 22;11(9):e0163122. doi: 10.1371/journal.pone.0163122 (PMC5033463; doi:10.1371/journal.pone.0163122)
Supplement: S1 Fig — (PDF) [file pone.0163122.s002.pdf]

S1 Figure. Geographic location of the study region in the Iberian Peninsula (small panel) and of the studied sites within the study area (large panel; coordinates in decimal degrees). The two red patches in the small panel denote the only records of *Flavipanurgus venustus* before the present study and the orange area denotes the probable species distribution according to IUCN (Patiny 2012). Dots in the large panel denote study sites and different colours denote the different studies and sampling types conducted in each site (see legend); note that in the two sites represented with green dots two different sampling types were conducted.

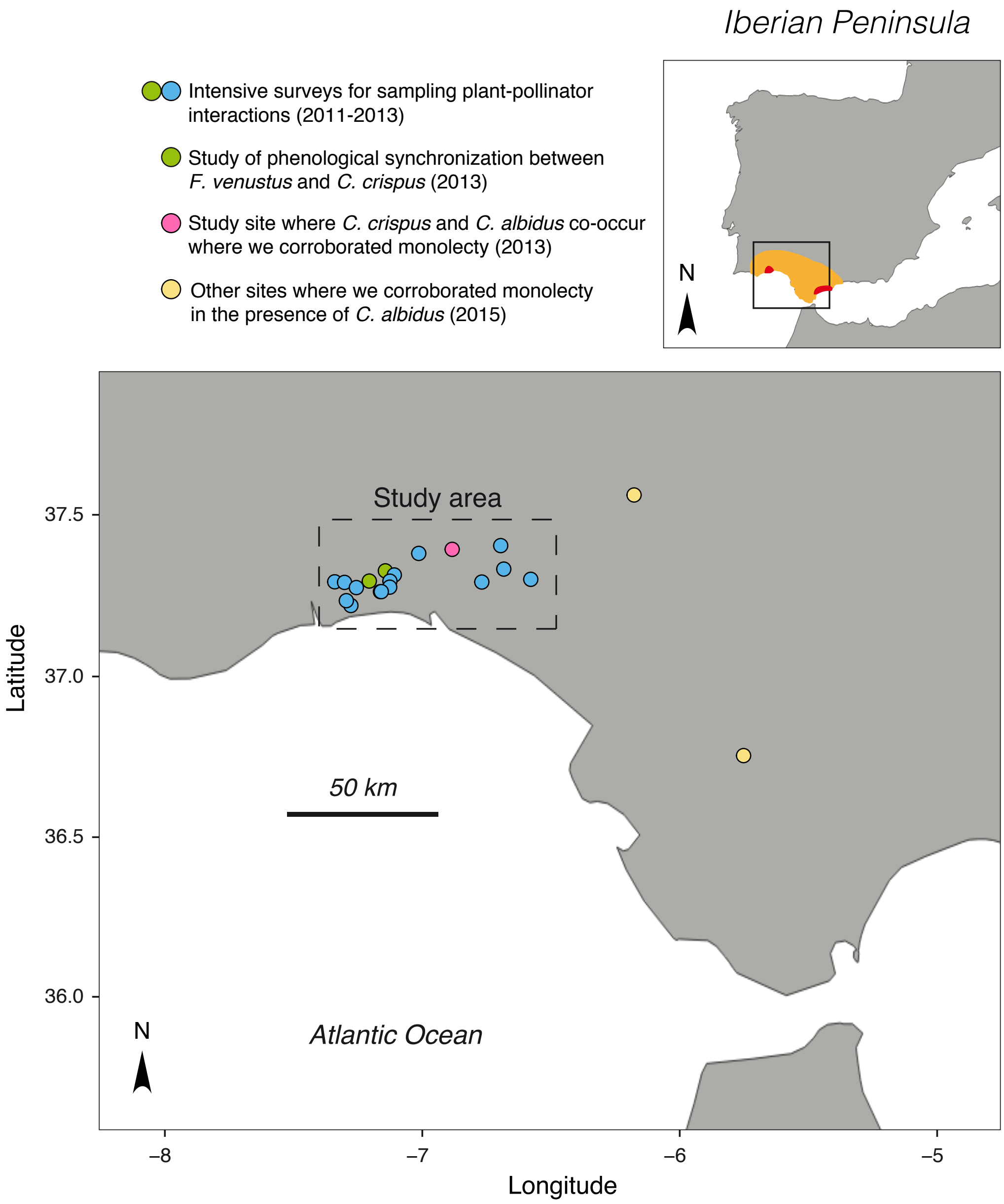

Patiny S (2012) *Atlas of the European Bees: genus Flavipanurgus.* STEP Project, Atlas Hymenoptera. Mons, Gembloux.
http://www.zoologie.umh.ac.be//hymenoptera/page.asp?ID=24. Date of access: 06/03/2016
http://maps.iucnredlist.org/map.html?id=13311712. Date of access: 06/03/2016
