# Supplementary material for: Total Bee Dependence on One Flower Species Despite Available Congeners of Similar Floral Shape
Source: PLoS One. 2016 Sep 22;11(9):e0163122. doi: 10.1371/journal.pone.0163122 (PMC5033463; doi:10.1371/journal.pone.0163122)

**S2 Figure.** (a) Flowers of the more abundant *Cistus* species in the studied woodland patches, the white-flowered *C. salviifolius* and *C. monspeliensis*, and the purple-flowered *C. crispus* (cd: corolla diameter); (b) Density of *Flavipanurgus venustus* bees (upper panel - line: median; box: quartiles; error bars: 10th/90th; circles: 5th/95th percentiles) and density of *Cistus* spp. flowers (bottom panel) in the studied woodland patches during the sampling periods 'March–April' and 'April–May'. The density of *C. crispus* flowers is shown in purple, whereas the density of other *Cistus* spp. is shown in white.

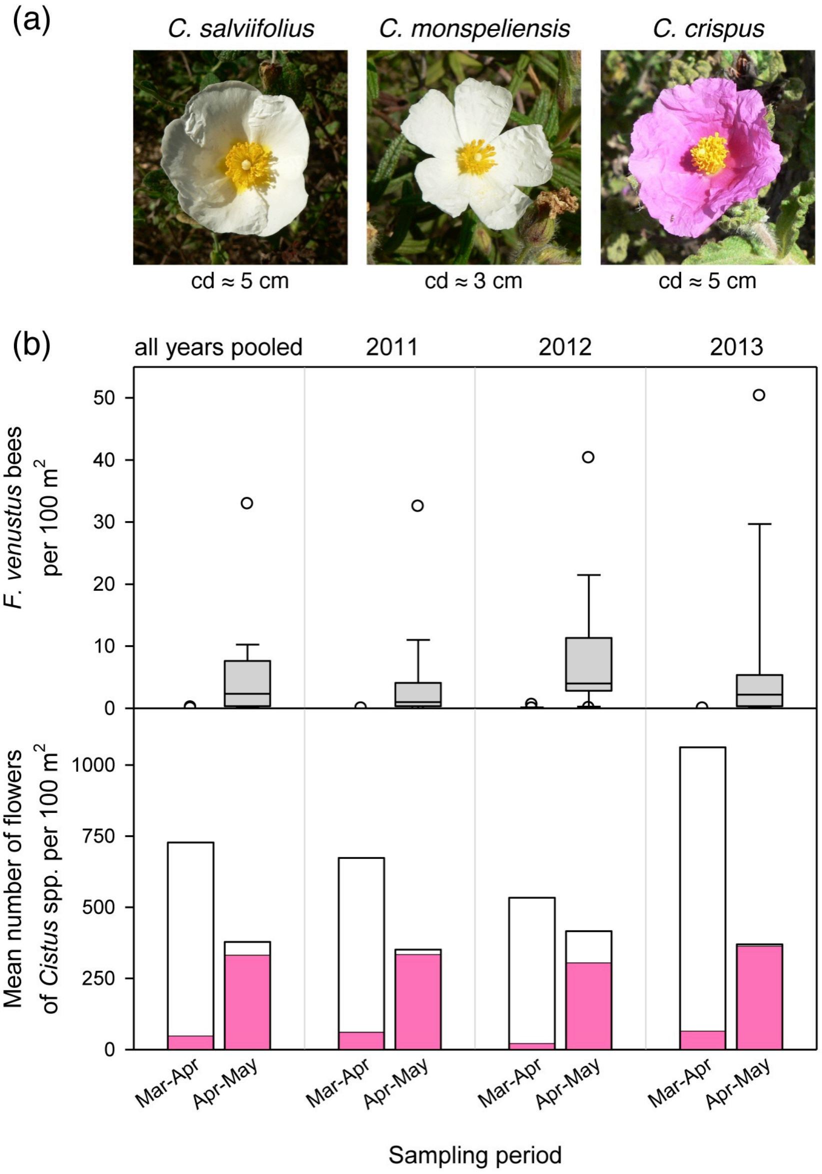

Supplement: S2 Fig — (PDF) [file pone.0163122.s003.pdf]
