# Supplementary material for: Total Bee Dependence on One Flower Species Despite Available Congeners of Similar Floral Shape
Source: PLoS One. 2016 Sep 22;11(9):e0163122. doi: 10.1371/journal.pone.0163122 (PMC5033463; doi:10.1371/journal.pone.0163122)
Supplement: S1 Table — (PDF) [file pone.0163122.s008.pdf]

S1 Table. Characteristics of the 17 study woodland patches studied. Each dot represents a sampling round; i.e. pollinators and flower-pollinator interactions were sampled along the two 1 m × 150 m fixed transects located within each patch. Blue dots: sampling rounds conducted during the period 'March–April'. Orange dots: sampling rounds conducted during the period 'April–May'.

	coordinates		§ patch size	§ woodland cover	*n* years	sampled years		
patch name	*x* UTM29S	*y* UTM29S	(ha)	within 1-km radius (%)	sampled	2011	2012	2013
La Barca*	660391.0	4129104.4	1.8	22.8	3	●●●●	●●●●	●●●●
Menajo*	665852.4	4132735.1	2.2	16.4	3	●●●●	●●●●	●●●●
Gibraleón	677224.0	4138920.6	7.3	14.9	3	●●●	●●●●	●●●●
Lucena	699212.5	4129622.6	5.8	27.3	3	●●●	●●●●	●●●●
Niebla	705343.1	4142232.1	4.9	11.6	3	●●●●	●●●●	●●●●
Pinar	667456.1	4127165.0	1.9	37.9	3	●●●●	●●●●	●●●●
Redondela	654200.5	4120601.8	3.6	9.7	3	●●●	●●●●	●●●●
Villablanca	648495.2	4128645.7	1.8	58.2	3	●●●	●●●●	●●●●
Bonares	706587.0	4134234.0	3.9	33.0	2	●●●	●●●●	
Cetrero	668957.3	4131399.0	7.5	17.1	2	●●●●	●●●●	
Estanque	652596.0	4122250.7	1.9	10.1	2	●●●●	●●●●	
Canal	667487.7	4129222.8	1.0	2.1	2		●●●●	●●●●
Chatarrero	664570.6	4125562.6	4.1	14.5	2		●●●●	●●●●
Cartaya	664323.3	4125588.8	1.9	17.1	1	●●●●		
Curva	651881.9	4128503.2	2.1	22.6	1	●●●●		
Gravera	655979.0	4126804.0	2.6	3.8	1	●●●●		
Rociana	716002.0	4130967.0	2.2	9.5	1	●●●		

* Sites chosen for phenological study.

§ Patch size and woodland cover within 1-km buffer radius were obtained from aerial digital orthophotos dating from 2011 using the GIS software ArcMap™ 10.0 (® ESRI).
