# Supplementary material for: Total Bee Dependence on One Flower Species Despite Available Congeners of Similar Floral Shape
Source: PLoS One. 2016 Sep 22;11(9):e0163122. doi: 10.1371/journal.pone.0163122 (PMC5033463; doi:10.1371/journal.pone.0163122)
Supplement: S2 Table — (PDF) [file pone.0163122.s009.pdf]

S2 Table. Observed occurrence of *Flavipanurgus venustus* bees in the 17 study woodland patches studied and the three study years. “●”: sampled along the belt transects; “●”: not sampled along belt transects but observed in the site; “–“: not sampled nor observed in the site.

	sampled years		
patch name	2011	2012	2013
La Barca*	●	●	●
Menajo*	●	●	●
Gibraleón	●	●	●
Lucena	–	●	●
Niebla	–	●	●
Pinar	●	●	●
Redondela	●	●	●
Villablanca	●	●	●
Bonares	●	●	
Cetrero	●	●	
Estanque	●	●	
Canal		●	●
Chatarrero		●	●
Cartaya	●		
Curva	●		
Gravera	●		
Rociana	–		

* Sites chosen for phenological study.
