# Supplementary material for: Total Bee Dependence on One Flower Species Despite Available Congeners of Similar Floral Shape
Source: PLoS One. 2016 Sep 22;11(9):e0163122. doi: 10.1371/journal.pone.0163122 (PMC5033463; doi:10.1371/journal.pone.0163122)
Supplement: S3 Table — (PDF) [file pone.0163122.s010.pdf]

S3 Table. List of the most common flower species (and family names) recorded across the study patches in the sampling periods 'May–April' and 'April–May' (total = 91 and 80 species belonging to 34 and 29 families, respectively). Only species recorded in at least five patches are shown. $n_{patches}$ = number of study patches each species was recorded along the sampling transects.

'March–April' period		'April–May' period	
Flower species	$n_{patches}$	Flower species	$n_{patches}$
Lavandula stoechas (Lamiaceae)	17	***Cistus crispus*** (Cistaceae)	17
Leontodon longirostris (Asteraceae)	16	*Galactites tomentosa* (Asteraceae)	17
Tuberaria sp (Cistaceae)	16	*Lavandula stoechas* (Lamiaceae)	17
Cistus crispus (Cistaceae)	15	*Leontodon longirostris* (Asteraceae)	17
Cistus ladanifer (Cistaceae)	14	*Elaeoselinum foetidum* (Apiaceae)	16
Cistus salvifolius (Cistaceae)	14	*Tolpis barbata* (Asteraceae)	16
Genista hirsuta (Fabaceae)	14	*Jasione montana* (Campanulaceae)	14
Echium plantagineum (Boraginaceae)	13	*Echium plantagineum* (Boraginaceae)	13
Galactites tomentosa (Asteraceae)	12	*Genista hirsuta* (Fabaceae)	12
Sonchus asper/oleraceus (Asteraceae)	12	*Tuberaria* sp. (Cistaceae)	12
Raphanus raphanistrum (Brassicaceae)	11	*Andryala integrifolia* (Asteraceae)	11
Anagallis arvensis (Primulaceae)	9	*Andryala ragusina* (Asteraceae)	11
Campanula lusitanica (Campanulaceae)	9	*Cistus salvifolius* (Cistaceae)	10
Cistus monspeliensis (Cistaceae)	9	*Halimium halimifolium* (Cistaceae)	10
Asteraceae sp.1 (Asteraceae)	8	Asteraceae sp.1 (Asteraceae)	9
Halimium halimifolium (Cistaceae)	8	*Cistus monspeliensis* (Cistaceae)	8
Tolpis barbata (Asteraceae)	8	*Sonchus asper/oleraceus* (Asteraceae)	8
Vicia sativa (Fabaceae)	8	*Crepis capillaris* (Asteraceae)	7
Asphodelus sp. (Xanthorrhoeaceae)	7	*Silene colorata* (Caryophyllaceae)	7
Linaria viscosa (Scrophulariaceae)	7	*Helichrysum picardii* (Asteraceae)	6
Muscari comosum (Liliaceae)	7	*Pulicaria odora* (Asteraceae)	6
Erodium sp. (Geraniaceae)	6	*Asphodelus* sp. (Xanthorrhoeaceae)	5
Linum bienne (Linaceae)	6	*Campanula lusitanica* (Campanulaceae)	5
Scorpiurus sp. (Fabaceae)	6	*Cistus ladanifer* (Cistaceae)	5
Antirrhinum orontium (Plantaginaceae)	5	*Convolvulus althaeoides* (Convolvulaceae)	5
Fabaceae sp.1 (Fabaceae)	5	*Linaria viscosa* (Scrophulariaceae)	5
Halimium calycinum (Cistaceae)	5	*Scabiosa columbaria* (Caprifoliaceae)	5
Leucojun trichophyllum (Amaryllidaceae)	5	*Ulex australis* (Fabaceae)	5
Oxalis pes-caprae (Oxalidaceae)	5	–	–
Rosmarinus officinalis (Lamiaceae)	5	–	–
Sesamoides canescens (Resedaceae)	5	–	–
