# Supplementary material for: Total Bee Dependence on One Flower Species Despite Available Congeners of Similar Floral Shape
Source: PLoS One. 2016 Sep 22;11(9):e0163122. doi: 10.1371/journal.pone.0163122 (PMC5033463; doi:10.1371/journal.pone.0163122)
Supplement: S4 Table — (PDF) [file pone.0163122.s011.pdf]

S4 Table. Standardized parameters (β) and statistics of the linear mixed models (sampling year as random factor) predicting the density of *F. venustus* bees (a) with the density of *Cistus crispus* flowers and also with the sampling date (Julian day); (b) with the variables included in 'M2' (i.e. flower density and sampling date; marked in yellow) together with patch size (area) and/or landscape woodland cover (% within a 1-km buffer radius); and (c) with variables included in 'M2' plus geographic coordinates (*x* and/or *y* UTM). Significant β-values – based on *t*-tests – are shown in bold-type (all $P < 0.001$). AICc: Akaike Information Criterion for small sample size; ΔAICc: difference between the AICc value of a model and that of the model with the lowest AICc; $R^2_{GLMM\ (m)}$: Marginal R^2 values (i.e. the variance explained by the fixed effects variables) of models were calculated following Nakagawa and Schielzeth (2013).

Models predicting local *F. venustus* density	β of predictor variables included in the model						model fit statistics		
	flower density	sampling date	patch size	woodland cover	longitude (*x*)	latitude (*y*)	AICc	ΔAICc	$R^2_{GLMM\ (m)}$
(a) flower density and sampling date									
M1	**0.312**	–	–	–	–	–	52.9	13.5	0.271
M2	**0.286**	**0.230**	–	–	–	–	41.3	1.9	0.479
(b) 'M2' + habitat fragmentation variables									
M3	**0.271**	**0.228**	–0.081	–	–	–	43.4	4.0	0.494
M4	**0.297**	**0.233**	–	0.048	–	–	43.9	4.5	0.465
M5	**0.280**	**0.230**	–0.074	0.035	–	–	46.3	6.9	0.477
(c) 'M2' + geographic coordinates									
M6	**0.245**	**0.205**	–	–	**–0.191**	–	39.4	0.0	0.583
M7	**0.277**	**0.227**	–	–	–	–0.108	42.8	3.4	0.501
M8	**0.244**	**0.204**	–	–	–0.206	0.024	42.4.	3.0	0.567

Results show that the abundance of *C. crispus* flowers significantly and consistently predicted the abundance of *F. venustus* bees when controlling by the sampling date, habitat loss and fragmentation, and the geographic location of the study patches (Table S2).
